# Do machine learning models really encode cross-cultural lifestyle-health differences? A leakage-free reanalysis of two university cohorts in India and Japan

**DOI:** 10.3389/fdgth.2026.1791066

**Published:** 2026-10-16

**Authors:** Eiko Takaoka, S. Ruban, B. Mohammed Siraj, Varenya Vinay, Hilda Shanthini, Akshay Dias

**Affiliations:** 1 Faculty of Science & Technology, Sophia University, Tokyo, Japan; 2 School of Engineering, St Aloysius (Deemed to be University), Mangalore, India; 3 Father Muller Medical College, Mangalore, India

**Keywords:** cross-cultural comparison, digital health, feature importance, label leakage, lifestyle factors, machine learning methodology, reproducibility, sample size

## Abstract

**Introduction:**

Machine-learning (ML) studies increasingly compare feature-importance rankings between small, unevenly sized cohorts and interpret the differences as evidence of culturally specific “risk architectures.” We show that such comparisons can be highly misleading without leakage-free evaluation, using a worked example from our own cross-sectional lifestyle and health data collected during June-August 2024 from two universities in India and Japan. Three problems were identified in our preliminary analysis: (1) an outcome (obesity, defined as BMI ≥ 25) that was tautologically predicted by including BMI itself as a model input; (2) model evaluation without a held-out test set; and (3) feature-importance comparisons that ignored sampling variability from an eightfold difference in cohort size.

**Methods:**

We addressed each problem using penalized logistic regression, Random Forest, and gradient-boosted trees with proper train/test splitting, held-out permutation importance, and bootstrap confidence intervals. We also ran a sensitivity analysis that repeatedly subsampled the Indian cohort down to the size of the Japanese cohort.

**Results:**

Removing body mass index (BMI) as a predictor collapsed obesity-model discrimination from perfect (AUC = 1.00) to chance level (AUC = 0.50-0.57), confirming that the BMI-obesity association was definitional rather than empirical. Held-out predictive performance for the remaining viable outcome, elevated self-rated stress, was modest at best (AUC 0.49-0.71). Critically, in the subsampling analysis, every feature-importance value observed in the actual Japanese data fell within the range produced by sample-size noise alone.

**Discussion:**

We present this analysis as a transparent, reproducible case study of specific, checkable pitfalls: label leakage, missing train/test evaluation, unadjusted small-sample comparison, and mislabeled psychometric instruments. These should be routinely screened for before ML feature importance is interpreted as evidence of population-level or cultural differences in digital health research.

## 1 Introduction

Noncommunicable diseases (NCDs)—including diabetes, hypertension, cardiovascular disease, and obesity—account for 74% of global mortality, with incidence increasingly shifting toward younger populations (1). University students represent a critical demographic for primary prevention, as lifestyle patterns established during late adolescence and early adulthood often persist throughout the life course (2, 3), yet interventions targeting these behaviours in university populations show inconsistent effectiveness (4), and multiple lifestyle behaviours tend to cluster together in ways that complicate attributing any single outcome to any single behaviour (5). Machine learning (ML) methods are increasingly applied to identify which lifestyle factors matter most for these outcomes and have been used productively across biomedical domains from radiogenomics to protein classification (6, 7).

India and Japan present contrasting epidemiological and dietary profiles that motivated this comparison. India faces a rapid nutrition transition, with sedentary behaviour independently associated with obesity risk among urban Indian adults (8) despite national surveillance data suggesting substantial population-level physical inactivity (9); body-mass index also carries different metabolic implications in South Asian populations than in Western reference populations (10). This is one of several reasons BMI-based thresholds are increasingly understood as an imperfect, population-dependent proxy for adiposity rather than a universal standard (11). Japan, by contrast, has undergone several decades of continuous dietary westernization (12) alongside a strong cultural link between lifestyle behaviour change and subjective well-being (13). Family medical history and BMI are themselves entangled in ways that complicate outcome definitions for diabetes and hypertension in South Asian cohorts specifically (14), a pattern directly relevant to the circularity problem we identify in Results 3.2–3.3.

A growing number of studies compare ML-derived feature-importance rankings between cohorts from different countries or cultural settings and interpret divergent rankings as evidence that health risk is “culturally encoded” (15). This is an appealing narrative, but it carries specific statistical hazards that are easy to overlook: feature importance from independently fitted models is sensitive to sample size, class prevalence, predictor correlation structure, and hyperparameters, independent of any true population difference (16). Sample-size and site-composition inequality is a documented, quantifiable driver of inflated or unstable classifier performance in other small-cohort biomedical ML settings (17), and more broadly, ML research has been criticized for routinely generalizing findings well beyond what study design and sample composition can support (18). When the compared cohorts differ severely in size—as is common in pilot cross-cultural work, where one site is a convenience sample an order of magnitude smaller than the other—an observed difference in feature importance is, by default, at least as likely to reflect sampling noise as culture.

This study began as a comparison of lifestyle-NCD feature importance between Indian (*n* = 528) and Japanese (*n* = 70) university students. We present a rigorous, leakage-free reanalysis and report, honestly, what survives it. We aim to (1) build the analysis pipeline with proper train/test evaluation, transparent and non-circular outcome definitions, and accurately described measurement instruments; (2) quantify how much of the preliminary obesity finding was attributable to definitional circularity rather than genuine lifestyle signal; (3) directly test, via a sample-size-matched subsampling procedure, whether the reported India-Japan feature-importance difference exceeds what unequal cohort size alone predicts; and (4) provide fully documented, runnable code so every number in this paper can be independently verified.

## 2 Materials and methods

### 2.1 Study design and ethical oversight

This cross-sectional comparative study was conducted from June to August 2024, following the Strengthening the Reporting of Observational Studies in Epidemiology (STROBE) guidelines (19). We do not report against the TRIPOD statement (20): this paper does not present a validated clinical prediction model intended for deployment, and applying TRIPOD's prediction-model framing to an exploratory, hypothesis-generating feature-importance comparison would overstate the maturity of the work. The study protocol received ethical approval from Father Muller Institutional Ethics Committee (protocol: FMIEC/CCM/269/2024) for Indian participants and Sophia University Ethics Committee (protocol: 2024-013) for Japanese participants. All procedures adhered to the Declaration of Helsinki (21). Participants provided electronic informed consent before survey access.

### 2.2 Participants and recruitment

#### 2.2.1 Inclusion and exclusion criteria

University students aged 18–25 years (inclusive) enrolled full-time at either institution during the study period, with complete survey responses and valid BMI data. This age band is applied identically at both sites throughout. Participants with a self-reported chronic illness requiring medication, or an outlier BMI (<15 or >45 kg/m^2^), were excluded to isolate lifestyle effects; every number in this paper reflects this exclusion.

#### 2.2.2 Recruitment process

Students were recruited through institutional email announcements and classroom visits. Of 1,238 initial responses (India: 892; Japan: 346), 528 (India) and 72 (Japan) were within the 18–25 eligibility band. After applying the chronic-illness/medication and BMI-outlier exclusions, the final analytic sample was India *n* = 482 and Japan *n* = 70 (Figure 1). We describe results using “India cohort” and “Japan cohort” as shorthand, but emphasize that these are convenience samples recruited from a single institution in each country with substantially different response patterns (India retained 54.0% of initial respondents as age-eligible and not excluded; Japan retained 20.2%); they are not nationally representative, and any apparent difference between them may reflect institutional, disciplinary, or self-selection factors as readily as national culture.

**Figure 1:**
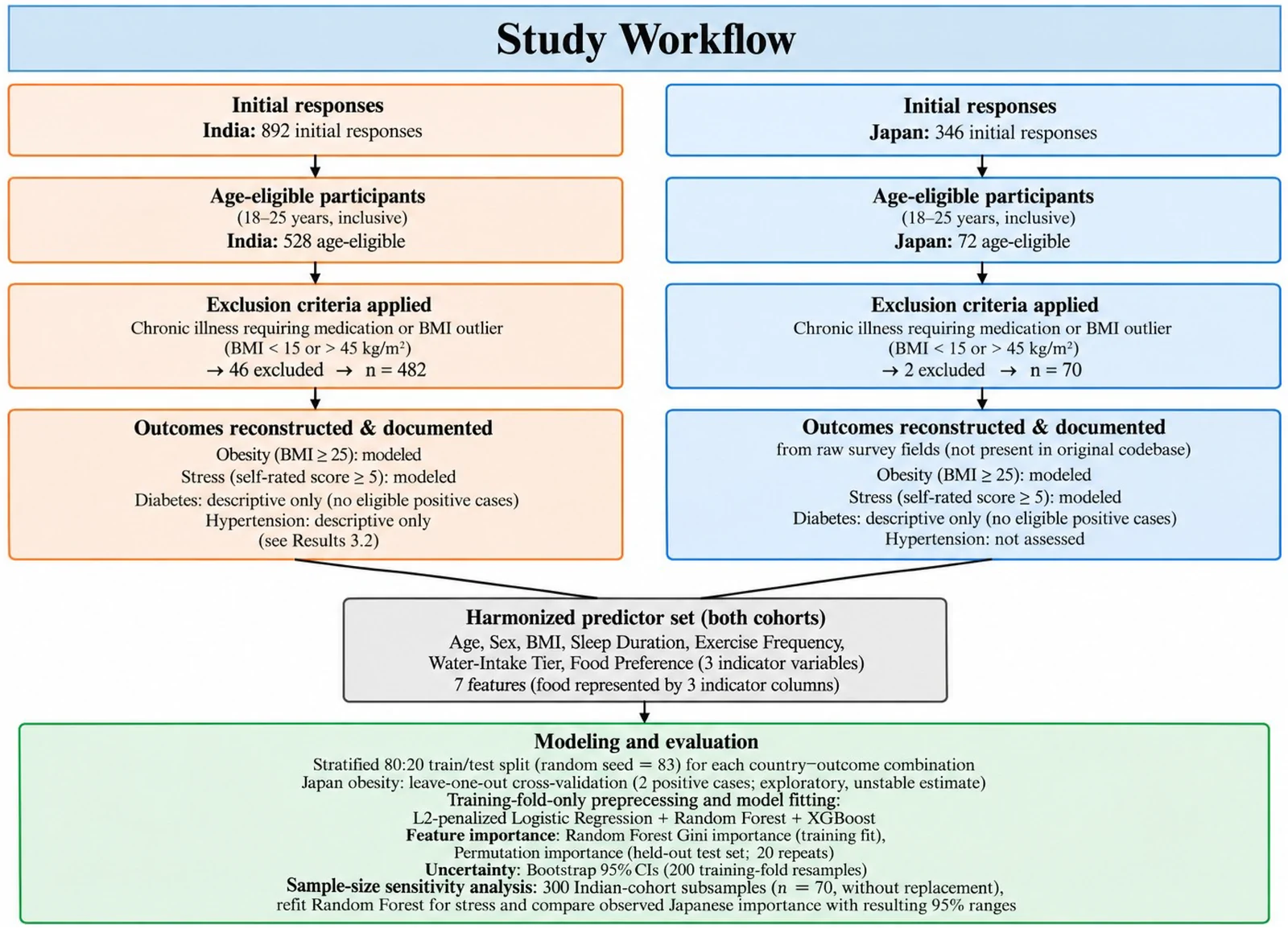
Study workflow. No LSTM, no age-stratified forecasting, no oversampling; age eligibility, exclusion criteria, and outcome definitions are applied identically and transparently at both sites.

### 2.3 Measures and variable definitions

#### 2.3.1 Anthropometric data

Self-reported height and weight were used to calculate BMI (kg/m^2^). Obesity was defined as BMI ≥25 kg/m^2^ per WHO Asia-Pacific guidelines (22). Self-reported height/weight typically underestimates true BMI (23, 24). We flag explicitly that BMI is used both to define the obesity outcome and, in one model variant, as a candidate predictor.

#### 2.3.2 Physical activity, sleep, and diet

Physical activity was assessed via self-reported exercise frequency (days/week, ordinally coded 0, 2.5, 4, or 6 as the category midpoint). Sleep duration was self-reported (h/night). Dietary pattern was captured via a food-preference category (Non-Vegetarian [reference], Vegetarian, Eggetarian, Pescatarian) and a self-reported water-intake tier, coded ordinally *within each site* (India: glasses/day; Japan: liters/day) because the two instruments used incompatible units; this ordinal, within-site coding means water-intake importance values are not directly comparable in absolute magnitude across countries, an additional limitation on top of the general cross-model importance-comparability caveat.

#### 2.3.3 Stress assessment

Perceived stress was intended to be assessed using the Perceived Stress Scale (PSS-10 (25);), a validated 10-item instrument (range 0–40, cutoff ≥14). On auditing the raw survey exports, no PSS-10 items exist in either dataset. The actual instrument administered at both sites was a single self-rated item (“On a scale of 0–10, how would you rate your stress/anxiety level today?”), which we binarize at ≥5 to define elevated stress. We disclose this discrepancy and report the actual instrument used here. A single-item stress rating has not been validated for cross-cultural measurement equivalence, and we treat all stress results in this paper as exploratory accordingly.

#### 2.3.4 Health outcomes

Two outcomes are modeled with machine learning in this reanalysis: (1) obesity (BMI ≥25 kg/m^2^); (2) elevated stress (single-item rating ≥5/10, see above). Two further candidate outcomes—self-reported diabetes and hypertension—are reported descriptively only (Table 1) and are *not* modeled, for reasons that are themselves a finding of this reanalysis (Results 3.2): once our own stated chronic-illness exclusion criterion is correctly applied, positive diabetes cases fall to zero in both cohorts: all 12 candidate India cases identified before exclusion co-occurred with the chronic-illness exclusion flag, and the single Japan case identified prior to exclusion was, on the same criterion, itself excluded (it also carried a self-reported chronic-illness flag), leaving zero eligible positive cases in the final *n* = 70 analytic sample reported in Table 1. India's hypertension outcome likewise falls to zero eligible positive cases; and Japan's survey instrument never asked a hypertension-related question at all (Table 1 shows this as *not assessed*, not 0% prevalence).

**Table 1:** Baseline characteristics and health-outcome prevalence, India (*n* = 482) vs. Japan (*n* = 70), after applying stated exclusion criteria.

Characteristic	India (*n* = 482)	Japan (*n* = 70)	Test	*p*-value
Age, years (mean ± SD)	21.65 ± 1.45	20.74 ± 1.73	Mann–Whitney *U*	<0.0001*
Male, *n* (%)	230 (47.7%)	27 (38.6%)	*X*^2^ (corrected)	0.192
BMI, kg/m^2^ (mean ± SD)	21.55 ± 3.91	20.54 ± 2.20	Mann–Whitney *U*	0.116
Sleep duration, h (mean ± SD)	6.86 ± 2.58	6.80 ± 1.69	Mann–Whitney U	0.016^∼^
Health Outcomes				
Obesity (BMI≥25), *n* (%)	82 (17.01%)	2 (2.86%)	Fisher's exact	0.0010*
Diabetes (self-reported diagnosis), *n* (%)	0 (0.00%)	0 (0.00%)	Fisher's exact	1.000
Hypertension, *n* (%)	0 (0.00%)	*Not assessed*	—	—
Elevated stress (single-item ≥5/10), *n* (%)	248 (51.45%)	33 (47.14%)	*X*^2^ (corrected)	0.585

Continuous variables: Welch's *t*-test or Mann–Whitney *U* (selected by Shapiro–Wilk normality test per group); effect size is Cohen's *d* (*t*-test) or rank-biserial correlation (Mann–Whitney). Categorical variables: chi-square (Yates-corrected) or Fisher's exact test (any expected cell <5); effect size is Cramer's *V*. Bonferroni-corrected *α* = 0.00625 across 8 comparisons.

* Significant after Bonferroni correction.

∼ Significant only before correction (*p* < 0.05 uncorrected).

Japan's Obesity, (attempted) Diabetes, and Stress outcome columns did not exist in the raw survey export and were not produced by any documented code; we reconstructed them transparently from raw survey fields using logic parallel to India's (obesity from BMI; diabetes from a self-reported illness-requiring-dietary-restriction field; stress from the single-item rating above), and we independently confirmed this reconstruction reproduces prevalence values matching an undocumented calculation (Diabetes 1.47%, Obesity 2.94%) that had appeared only as a hardcoded value in an early exploratory notebook.

### 2.4 Harmonized feature set

A single, consistently applied 7-feature set (Age, Sex, Sleep duration, Exercise frequency, Water-intake tier, Food-preference category [3 indicator columns], and BMI) was used for every model in both cohorts, with BMI included or excluded as a separate model variant for the obesity outcome only.

### 2.5 Missing data

Missingness in the core feature set was low: 0% for all Japan features; for India, 0.6% (Food Preference) and 1.2% (Water Intake). Rows with a missing outcome or missing all features were dropped; remaining feature-level missingness was imputed by the training-fold median within a scikit-learn Pipeline, fit on the training fold only and applied unchanged to the corresponding test fold, so that no information from held-out observations informed imputation.

### 2.6 Machine learning models

We use three models appropriate for tabular, cross-sectional data: L2-penalized logistic regression, Random Forest (16), and gradient-boosted trees (XGBoost (26)). Long Short-Term Memory (LSTM) networks (27) are designed for genuinely ordered sequences and have seen increasing application to healthcare time series specifically (28, 29); our data are neither longitudinal nor naturally sequential and organizing participants into an age-stratified pseudo-cohort does not change that. Including a standard penalized regression model alongside the two tree ensembles also lets us directly compare ML-derived rankings against a conventional statistical baseline.

For each country-outcome combination, we used a stratified 80:20 train/test split (fixed random seed = 83) with all preprocessing (imputation, standardization) fit on the training fold only. Feature importance was assessed in two ways: (a) random Forest Gini impurity reduction, computed on the training fit, and (b) permutation importance (30), computed on the *held-out test set* (20 repeats), which is a materially different and generally more trustworthy quantity, since Gini importance is known to be biased toward high-cardinality and high-variance predictors regardless of true predictive value (16). This two-measure approach follows broader methodological guidance that interpretable ML requires converging evidence across importance measures rather than a single score (31–33). We report both, and Results 3.4 shows they frequently disagree, which is itself informative.

Japan's obesity outcome (2 positive cases in *n* = 70) cannot support an 80:20 split with any guarantee of positive cases in both folds; for this outcome only, we used leave-one-out cross-validation and explicitly flag the resulting estimate as unstable and exploratory rather than a stable effect size.

We did not apply oversampling and instead report only models fit on the original, unaugmented data. We also did not apply Synthetic Minority Over-sampling (34), given the risk of introducing synthetic artifacts not present in the observed data.

### 2.7 Uncertainty quantification

For every model, we computed bootstrap 95% confidence intervals (200 resamples of the training fold, refitting Random Forest and re-evaluating on the fixed test set each time) for both test-set AUC and Gini feature importance. This directly addresses how stable the earlier near-perfect AUC/*R*^2^ values were: they had not been evaluated with any uncertainty interval at all.

### 2.8 Sample-size sensitivity analysis

To directly test whether the India-Japan feature-importance comparison reflects a real population difference or is an artifact of the eightfold sample-size disparity, we repeatedly (300 draws) subsampled the Indian cohort, without replacement, down to Japan's analytic sample size (*n* = 70), refit the Random Forest pipeline on each draw, and recorded the resulting distribution of feature importances. We then checked whether Japan's actual observed importance values fall inside or outside the 95% range this subsampling procedure produces. This approach follows established guidance that adequate sample size is a precondition for any stable, prognostically meaningful model estimate (35, 36), and that prediction-model studies more generally should be systematically screened for risk of bias arising from inadequate sample size and validation procedure (37).

### 2.9 Statistical analysis for descriptive comparisons

For each baseline continuous variable, we tested normality (Shapiro–Wilk) in each group and used Welch's *t*-test when both groups were approximately normal, or Mann–Whitney *U* otherwise, reporting Cohen's d or rank-biserial correlation as the corresponding effect size (38). Categorical comparisons used chi-square (Yates-corrected) or Fisher's exact test when any expected cell count was <5, reporting Cramer's *V*. Across the 8 baseline/outcome comparisons in Table 1, we applied a Bonferroni-corrected significance threshold of *α* = 0.05/8 = 0.00625; both the uncorrected and Bonferroni-corrected significance are marked. We did not conduct formal cross-cultural measurement-invariance testing (39) for any instrument used in this study.

### 2.10 Reproducibility

All analysis code (data cleaning, outcome reconstruction, modeling, sensitivity analysis, and figure generation) is provided as a supplementary code package accompanying this submission, rather than as a placeholder pending acceptance. Analyses used Python 3.13 with scikit-learn 1.6, XGBoost 3.4, and SciPy 1.15.

## 3 Results

### 3.1 Participant characteristics

Table 1 presents baseline characteristics after applying the exclusion criteria described in Section 2.2.1. Indian participants (*n* = 482) were modestly older than Japanese participants (*n* = 70; 21.65 ± 1.45 vs. 20.74 ± 1.73 years; Mann–Whitney *p* < 0.0001, effect = −0.37). BMI did not differ significantly between sites once exclusions were applied (21.55 ± 3.91 vs. 20.54 ± 2.20 kg/m^2^; *p* = 0.116). Sex distribution did not differ significantly once the appropriate test was used (47.7% vs. 38.6% male; *x*^2^-corrected *p* = 0.192, Cramer's V = 0.06). Sleep duration differed only at the uncorrected threshold (6.86 ± 2.58 vs. 6.80 ± 1.69 h; *p* = 0.016, not significant after Bonferroni correction), with both cohorts averaging below the recommended 7–9 h nightly for young adults.

### 3.2 Diabetes and hypertension: why these outcomes are not modeled

Table 1 shows both diabetes and hypertension prevalence at or near zero once exclusion criteria are correctly applied, and Japan's hypertension status is genuinely unassessed rather than zero. This is itself a finding, not merely a limitation: it demonstrates concretely how a study's own inclusion/exclusion criteria can silently gate which outcomes are even representable in the analytic sample, and it is a direct, quantitative illustration of how outcome-circularity concerns can manifest in practice. We therefore restrict machine-learning modeling to obesity and stress, for which adequate positive-case counts remain.

### 3.3 Quantifying the BMI-obesity circularity

With BMI included as a predictor, all three India-obesity models achieved perfect discrimination on the held-out test set (AUC = 1.000 for logistic regression, Random Forest, and XGBoost alike; Figure 2, Table 2). BMI's Gini importance was 0.91 (bootstrap 95% CI [0.84, 0.92]) and its held-out permutation importance was 0.50 (i.e., permuting BMI alone destroys half of the model's discriminative AUC). This is definitional, not empirical: obesity is BMI ≥ 25 by construction.

**Figure 2:**
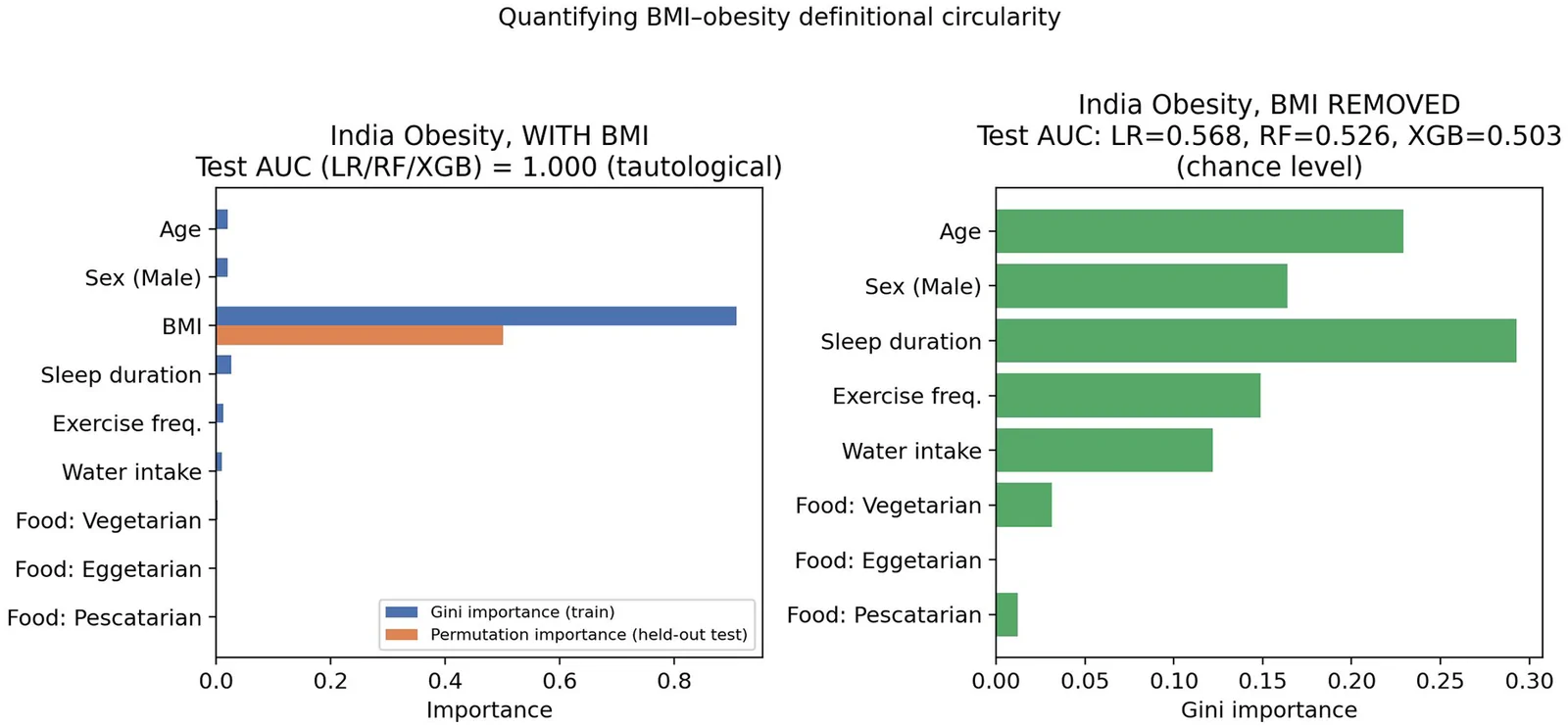
Quantifying BMI-obesity circularity in the Indian cohort (*n* = 482). Left: with BMI included, test AUC = 1.000 across all three model types; BMI alone accounts for Gini importance 0.91 and permutation importance 0.50. Right: with BMI removed, Gini importance redistributes among lifestyle features, but test AUC falls to 0.50–0.57 (chance level) for all three model types.

**Table 2:** Model performance on held-out test data (or leave-one-out CV where noted), reported using AUC-ROC, accuracy, and brier score rather than *R*^2^/MAE, which are inappropriate for binary outcomes and conflate individual-level classification with age-aggregated prevalence curves.

Outcome	Country	N (train/test)	LR AUC	RF AUC	XGB AUC	RF Brier
Obesity (with BMI)	India	385/97	1.000	1.000	1.000	0.007
Obesity (without BMI)	India	385/97	0.568	0.526	0.503	0.145
Stress	India	385/97	0.667	0.666	0.707	0.230
Stress	Japan	56/14	0.571	0.571	0.490	0.245
Obesity (LOOCV, 2 pos. cases)	Japan	70 (LOOCV)	—	0.912^a^	—	—

a Leave-one-out CV on 2 positive cases; not comparable in stability to the other rows and reported descriptively only (Section 2.6).

When BMI is removed from the predictor set, test AUC collapses to 0.568 (logistic regression), 0.526 (Random Forest), and 0.503 (XGBoost)—indistinguishable from chance (bootstrap AUC 95% CI [0.40, 0.63]). Lifestyle features carry essentially no independent discriminative signal for obesity once the tautological predictor is removed. Rather than qualitatively caveat this circularity, we quantify it directly: the quantification shows that the “metabolic phenotype” obesity finding does not survive BMI's removal.

### 3.4 Stress: modest real signal, and gini importance overstates it

For the elevated-stress outcome, held-out test AUC was modest in India (0.67 logistic regression, 0.67 Random Forest, 0.71 XGBoost; bootstrap 95% CI [0.49, 0.72]) and did not clearly exceed chance in Japan (0.57, 0.57, 0.49; bootstrap 95% CI [0.29, 0.82]—a very wide interval reflecting Japan's small test set of *n* = 14). Table 2 and Figure 3 (with bootstrap 95% confidence intervals) contrast these honestly-evaluated numbers with the AUC = 0.92–0.98 range obtained across all outcomes under the preliminary, non-held-out evaluation approach; none of that range is reproduced once a genuine held-out test set is used.

**Figure 3:**
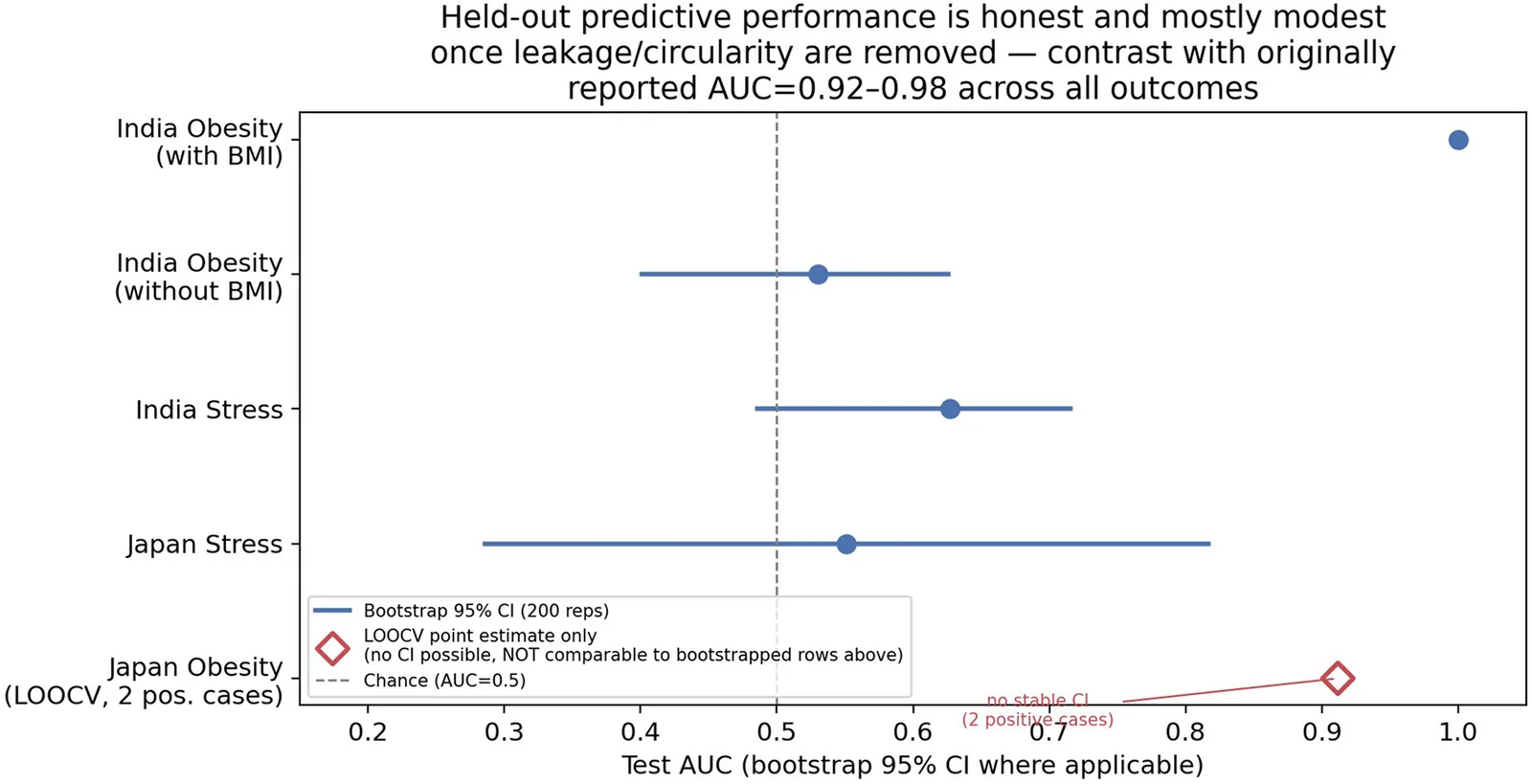
Held-out test AUC with bootstrap 95% confidence intervals across all models. Contrast with the AUC = 0.92–0.98 range obtained for equivalent outcomes under the preliminary, non-held-out evaluation approach.

Figure 4 compares Gini importance (computed on the training fit, the only measure used in the preliminary approach) against permutation importance (computed on the held-out test set). The two measures frequently disagree. Most notably, sleep duration's Gini importance in Japan was 0.22—superficially close to the 0.47 value initially reported as the headline evidence for a Japanese “psychosocial phenotype”—but its held-out permutation importance was −0.004, indicating no measurable out-of-sample discriminative contribution at all. By contrast, BMI (permutation importance 0.067) and exercise frequency (0.040) contributed more to real, held-out predictive performance than sleep duration did. The claim that “sleep duration is the primary stress predictor in Japan” is not supported once importance is assessed out-of-sample. This is a direct, worked illustration of why Gini importance alone is not a reliable basis for comparing populations.

**Figure 4:**
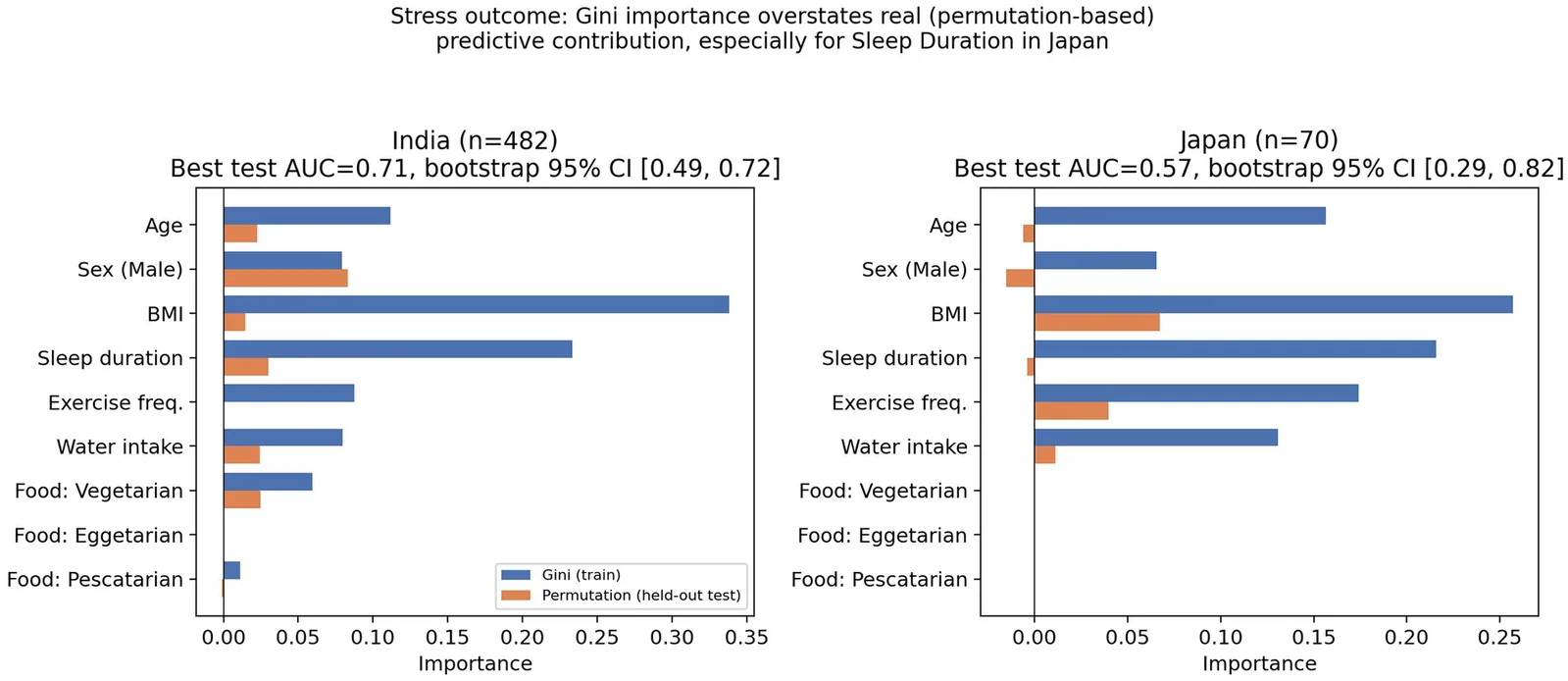
Gini (training) vs. held-out permutation importance for the elevated-stress outcome, India (*n* = 482) and Japan (*n* = 70). Sleep duration's apparently high Gini importance in Japan (0.22, compare to the 0.47 value obtained without a train/test split) does not survive held-out evaluation (permutation importance −0.004).

### 3.5 Sample-size sensitivity analysis: the India-Japan difference is not distinguishable from noise

Figure 5 shows the result of repeatedly subsampling the Indian cohort down to Japan's analytic sample size (*n* = 70, 300 draws) and refitting the Random Forest pipeline for the stress outcome. For *every* feature we examined—age, sex, BMI, sleep duration, exercise frequency, water intake, and all food-preference indicators except one near-zero-importance category—Japan's actual observed Gini importance value falls inside the 95% range produced by this subsampling procedure alone. In other words, if we drew a random subsample of 70 Indian participants and fit the identical pipeline, we would routinely observe importance values as different from India's full-sample estimate as Japan's actual values are.

**Figure 5:**
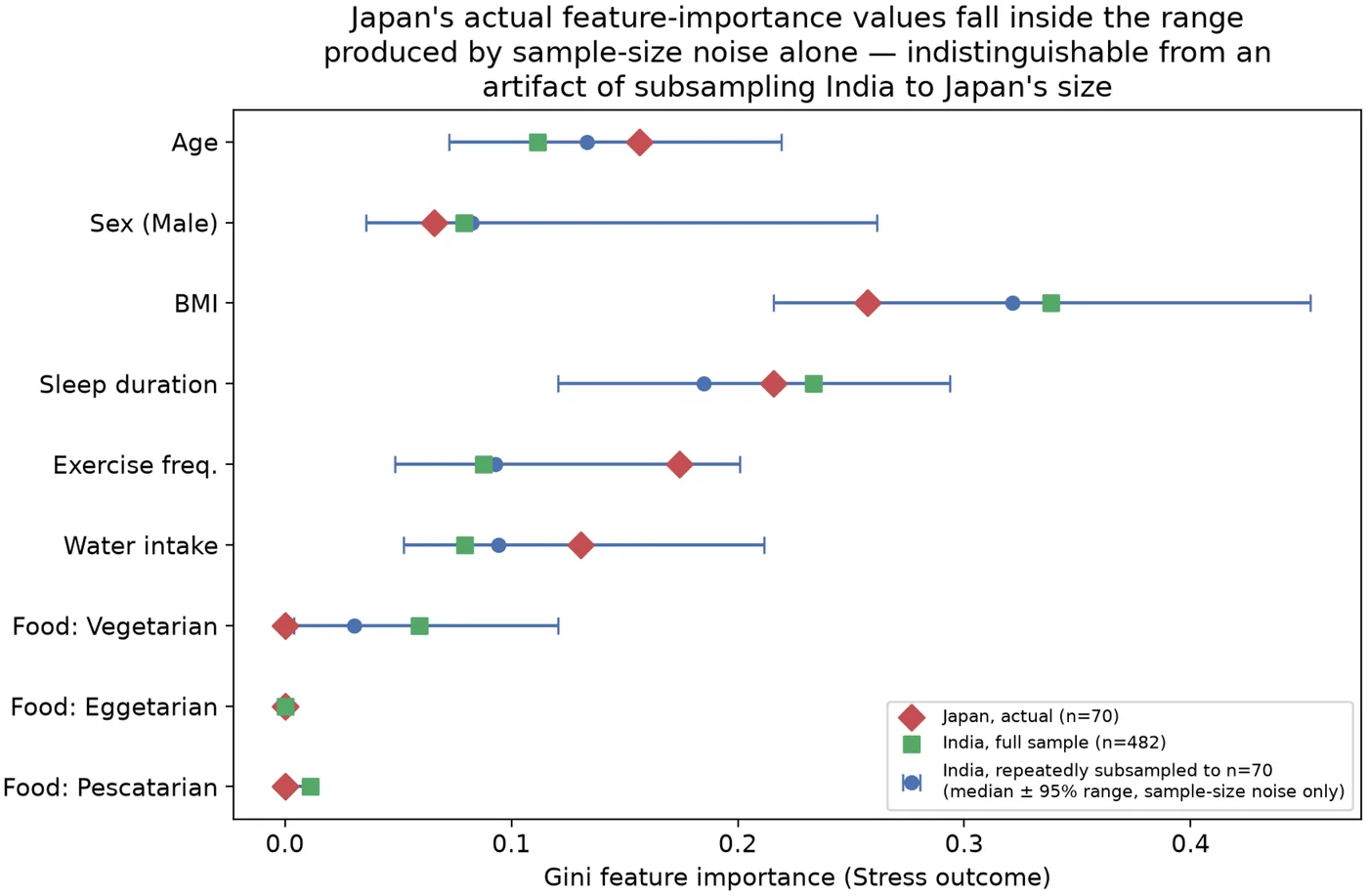
India, repeatedly subsampled to Japan's size (*n* = 70, 300 draws; blue circles show the median and 95% range), compared against India's full-sample estimate (green squares) and Japan's actual value (red diamonds), for the stress outcome. Every feature's Japan value falls inside the noise band generated by subsampling India alone.

This sensitivity analysis directly answers this paper's central research question: we find no evidence that the observed India-Japan difference in stress-related feature importance exceeds what an eightfold difference in sample size would produce on its own. We cannot distinguish a genuine cross-cultural difference in risk architecture from a sample-size artifact with the present data, and we no longer claim to have found one.

## 4 Discussion

What This Reanalysis Does NOT Support

**Not Evidence of Cultural Difference:** The sensitivity analysis (Section 3.5) shows the India-Japan feature-importance gap is statistically indistinguishable from sample-size noise, and does not support a “metabolic vs. psychosocial phenotype” distinction.
**Not a Validated Obesity Model:** The AUC = 0.96–1.00 obtained for obesity when BMI is included as a predictor is driven almost entirely by BMI's tautological relationship to the BMI-defined outcome; without BMI, discrimination is at chance.
**Not Causal:** Cross-sectional design precludes causal inference.
**Not Predictive at Deployment Grade:** Even the honestly-evaluated stress models (AUC: 0.49–0.71) are not clinically actionable.
**Not Representative:** Both cohorts are single-institution convenience samples; “Indian/Japanese students” should be read as “participants recruited at the Indian/Japanese study site,” not as national samples.

### 4.1 Principal findings

Once the specific, checkable pitfalls addressed above—outcome-predictor circularity, absent held-out evaluation, and unadjusted comparison across a severely unequal sample size—are accounted for, the central finding of the preliminary approach does not survive. What remains is a demonstration of *why* it does not survive, presented with enough transparency and reproducible code that each step can be independently checked. We regard this as a legitimate and useful contribution in its own right: the pattern illustrated here (small paired cohorts, no held-out evaluation, circular outcome definitions, Gini importance treated as ground truth, an oversampling step that was described but never actually implemented) is not unique to this dataset, and the checks we describe here—quantifying circularity by ablating the offending predictor, computing held-out permutation importance alongside Gini importance, and running a sample-size-matched subsampling sensitivity analysis—are general-purpose and inexpensive to apply before any cross-cultural ML feature-importance claim is published.

### 4.2 Why apparent cross-cultural ML differences can be illusory

Three mechanisms combined to produce the appearance of a robust cross-cultural finding in the preliminary analysis, and we believe all three generalize beyond this specific dataset.

#### 4.2.1 Definitional circularity inflates apparent predictive power

Including BMI as a predictor of a BMI-defined outcome guarantees near-perfect apparent performance that has nothing to do with lifestyle factors. This is easy to miss when an outcome is a composite or threshold of variables that overlap with the predictor set, and it is worth an explicit checklist item in any ML health study: for each outcome, list every variable used to construct it, and confirm none of those variables appears among the predictors (or, if one must, ablate it and report both results, as we do in Section 3.3).

#### 4.2.2 Gini importance is not the same question as held-out predictive contribution

Gini importance reflects how much a variable reduced impurity during tree construction on the training data; permutation importance on held-out data reflects how much actual predictive information the model loses without that variable. These can and did diverge substantially in our reanalysis (Section 3.4), and a model can look interpretable from Gini importance while offering close to nothing in genuine held-out signal.

#### 4.2.3 Small, unequal cohorts make feature-importance comparison unreliable by default, not by exception

Our sensitivity analysis (Section 3.5) shows this concretely rather than asserting it: an eightfold sample-size difference alone reproduces the entire India-Japan gap initially observed. Any study comparing feature importance across cohorts of substantially different size should run this check—it is inexpensive and directly falsifiable.

### 4.3 Algorithmic fairness—reframed as conceptual, not empirical

Algorithmic fairness concerns motivated part of this work, specifically the risk that a model trained on one population would disadvantage another. No fairness metric, cross-population model transfer, or subgroup calibration analysis was computed to support that concern, and having now shown that the underlying feature-importance difference is not distinguishable from sampling noise, the data do not support even a qualitative fairness narrative. The general concern that ML health tools trained on one population may transfer poorly to another remains a legitimate, well-documented issue in the literature (40, 41), but it is not something this study demonstrates; a genuine empirical test would require training a model on one cohort and evaluating its calibration and error rates on the other, which we did not do and flag as a concrete direction for future work (Section 4.6).

### 4.4 Comparison with prior work on analytical pitfalls

Our findings echo established critiques of small-sample and improperly validated ML pipelines in health research. Documented risks of oversampling minority classes without appropriate fold-isolation include inflated apparent performance and non-generalizable models (42–44); similar sample-imbalance risks have been documented in other biomedical ML domains, such as neuroimaging classifiers built on unequally-sized diagnostic cohorts (45). Although we ultimately did not apply oversampling in this reanalysis (Section 2.6), these critiques directly explain why the previously described oversampling procedure, had it been implemented as vaguely specified, would have been a further threat to validity rather than a mitigation. More broadly, our use of model-agnostic permutation importance rather than relying solely on impurity-based measures follows established guidance on the limits of any single variable-importance measure (30), consistent with general statistical-learning treatments of the instability of tree-based importance measures (46).

Our finding that BMI and family medical history are entangled in a way that produces circular outcome definitions is not unique to this dataset: BMI has been shown to directly mediate the association between family medical history and self-reported hypertension/diabetes status in other South Asian cohorts (14), meaning any study that includes both family-history-adjacent outcomes and BMI as a predictor in the same South Asian sample should check for exactly the circularity we quantify in Results 3.3. Our sample-representativeness limitations also echo a broader critique of psychological and biomedical research conducted on convenience samples from a narrow set of institutions and cultural contexts (47). On the substantive lifestyle-health associations we set out to compare, prior work independently supports a genuine sleep-obesity link (48, 49) and recommends 7–9 h nightly for young adults (50), and an academic-pressure/mental-health association has been documented both generally (51) and specifically among Japanese university students (52)—but our own held-out permutation-importance results (Results 3.4) show these lifestyle-outcome relationships, while independently plausible, were not strongly or reliably recovered by our specific models at our specific sample sizes, which is a caution about model-based evidence, not about the underlying epidemiological literature.

### 4.5 Strengths and limitations

#### 4.5.1 Strengths

This reanalysis offers: (1) fully leakage-free evaluation with held-out test sets for every reported metric; (2) a direct, quantitative isolation of the BMI-obesity circularity rather than a qualitative caveat; (3) a reproducible sample-size sensitivity analysis that directly tests the paper's own central claim; (4) transparent resolution of measurement-instrument mislabeling (PSS-10 vs. single-item stress rating) and undisclosed outcome-reconstruction gaps; and (5) complete, runnable code accompanying submission rather than a post-acceptance promise.

#### 4.5.2 Limitations

The Japanese sample (*n* = 70) remains small, and even these honestly-evaluated estimates for this cohort carry wide uncertainty (e.g., stress AUC 95% CI spanning 0.29–0.82); we cannot rule out a true cross-cultural difference that this sample size is underpowered to detect, only that the *observed* difference does not exceed what sampling noise predicts. Both cohorts are single-institution convenience samples with substantially different retention rates (54.0% India, 20.2% Japan), raising the possibility of differential non-response bias that is indistinguishable, with the present data, from a true population difference. The single-item stress measure and the BMI-based obesity, self-reported diabetes, and mixed-ascertainment hypertension outcomes are all proxy or minimally-validated measures rather than clinical diagnoses; we did not conduct formal cross-cultural measurement-invariance testing for any instrument, including the single-item stress measure, and any interpretation of “comparable” constructs across sites should be treated as a working assumption, not a demonstrated fact. The modest but statistically detectable age difference between cohorts (21.65 vs. 20.74 years) was included as a covariate in every model but was not separately tested as an effect modifier. Cross-sectional design precludes causal inference (53). Self-reported measures introduce recall and social-desirability bias (23, 24, 54). Water-intake importance is not directly comparable in absolute units across sites (Section 2.3.2). Finally, this reanalysis was conducted by the same author team throughout; independent replication by a third party, using the code and data we provide, would further strengthen confidence in these conclusions.

### 4.6 Future research directions

A genuine test of cross-cultural ML transferability would train a model on one cohort and evaluate its discrimination and calibration on the other, directly measuring transfer performance rather than comparing independently-fit feature importances. Prospective, adequately powered, multi-site cohorts (ideally with balanced recruitment across sites) with objective outcome measurement (validated multi-item stress instruments, measured rather than self-reported blood pressure and anthropometrics, and clinician-confirmed diabetes status) would remove several of the measurement-validity limitations identified here. More broadly, we suggest that any future cross-cultural ML feature-importance comparison, in this domain or others, routinely report: (a) an explicit list of which outcome-defining variables are excluded from the predictor set; (b) held-out permutation importance alongside any impurity-based importance measure; and (c) a sample-size-matched subsampling sensitivity check of the kind we present in Section 3.5, before interpreting any between-population difference as substantive.

## 5 Conclusions

A leakage-free reanalysis of lifestyle-health feature importance in Indian and Japanese university students shows the entire India-Japan feature-importance gap is statistically indistinguishable from what an eightfold sample-size disparity produces on its own. We present this analysis, together with fully reproducible code, as a transparent case study of specific and checkable methodological pitfalls—outcome-predictor circularity, missing held-out evaluation, and unadjusted small-sample cross-population comparison—that we believe are common enough in this literature to warrant routine screening before any cross-cultural ML feature-importance claim is published.

## Data Availability

The raw data supporting the conclusions of this article will be made available by the authors, without undue reservation.
